# Preliminary Virtual Screening Studies to Identify GRP78 Inhibitors Which May Interfere with SARS-CoV-2 Infection

**DOI:** 10.3390/ph13060132

**Published:** 2020-06-25

**Authors:** Andreia Palmeira, Emília Sousa, Aylin Köseler, Ramazan Sabirli, Tarık Gören, İbrahim Türkçüer, Özgür Kurt, Madalena M. Pinto, M. Helena Vasconcelos

**Affiliations:** 1 Laboratory of Organic and Pharmaceutical Chemistry (LQOF), Department of Chemical Sciences, Faculty of Pharmacy, University of Porto, Rua de Jorge Viterbo Ferreira, 228, 4050-313 Porto, Portugal; andreiapalmeira@gmail.com (A.P.); esousa@ff.up.pt (E.S.); madalenakijjoa@gmail.com (M.M.P.); 2 Interdisciplinary Centre of Marine and Environmental Research (CIIMAR), 4450-208 Matosinhos, Portugal; 3 Department of Biophysics, Pamukkale University Faculty of Medicine, 20190 Denizli, Turkey; 4 Department of Emergency Medicine, Kafkas University Faculty of Medicine, 36000 Kars, Turkey; ramazan_sabirli@hotmail.com; 5 Department of Emergency Medicine, Pamukkale University Faculty of Medicine, 20190 Denizli, Turkey; tarikgoren92@hotmail.com (T.G.); iturkcuer@pau.edu.tr (İ.T.); 6 Department of Microbiology, Acibadem Mehmet Ali Aydinlar University School of Medicine, 34752 Istanbul, Turkey; ozgur.kurt@acibadem.edu.tr; 7 Instituto de Investigação e Inovação em Saúde (i3S), University of Porto, 4200-135 Porto, Portugal; 8 Cancer Drug Resistance Group, Institute of Molecular Pathology and Immunology of the University of Porto (IPATIMUP), 4200-135 Porto, Portugal; 9 Laboratory of Microbiology, Department of Biological Sciences, Faculty of Pharmacy, University of Porto, 4050-313 Porto, Portugal

**Keywords:** Covid-19, SARS-CoV-2, GRP78, gene expression, virtual screening, antiviral, repurposed drugs

## Abstract

SARS-CoV-2 Spike protein was predicted by molecular docking to bind the host cell surface GRP78, which was suggested as a putative good molecular target to inhibit Covid-19. We aimed to confirm that GRP78 gene expression was increased in blood of SARS-CoV-2 (+) versus SARS-CoV-2 (−) pneumonia patients. In addition, we aimed to identify drugs that could be repurposed to inhibit GRP78, thus with potential anti-SARS-CoV-2 activity. Gene expression studies were performed in 10 SARS-CoV-2 (−) and 24 SARS-CoV-2 (+) pneumonia patients. A structure-based virtual screen was performed with 10,761 small molecules retrieved from DrugBank, using the GRP78 nucleotide binding domain and substrate binding domain as molecular targets. Results indicated that GRP78 mRNA levels were approximately four times higher in the blood of SARS-CoV-2 (+) versus SARS-CoV-2 (−) pneumonia patients, further suggesting that GRP78 might be a good molecular target to treat Covid-19. In addition, a total of 409 compounds were identified with potential as GRP78 inhibitors. In conclusion, we found preliminary evidence that further proposes GRP78 as a possible molecular target to treat Covid-19 and that many clinically approved drugs bind GRP78 as an off-target effect. We suggest that further work should be urgently carried out to confirm if GRP78 is indeed a good molecular target and if some of those drugs have potential to be repurposed for SARS-CoV-2 antiviral activity.

## 1. Introduction

Coronaviruses mainly cause enzootic infections in birds and mammals but in some cases have been capable of crossing the species barrier and infect humans [1]. Indeed, since 2002, β-coronaviruses have caused three zoonotic outbreaks [1]. The Coronaviridae family includes the Severe Acute Respiratory Syndrome (SARS) virus (SARS-CoV), the Middle East Respiratory Syndrome (MERS) virus (MERS-CoV), and the recently found Severe Acute Respiratory Syndrome Coronavirus 2 (SARS-CoV-2) [2]. The SARS-CoV-2 emerged as a zoonotic outbreak at the end of 2019, causing a disease named Covid-19 and being responsible for a pandemic within very few months [1].

SARS-CoV-2 is an enveloped virus with a positive-sense, single-stranded RNA genome [2]. This virus enters host cells by receptor-mediated endocytosis [2]. The spike S glycoprotein allows the attachment and virus internalization to the host cell [3], by binding to the host ACE2 receptor [4,5,6,7]. Cell entry also depends on the host cellular serine protease TMPRSS2 [8,9]. SARS-CoV-2 then uses the host cell’s machinery to produce more virus. The new viruses are assembled, enveloped and released from the cells via exocytosis, to infect other cells [9]. 

Viral glycoproteins from other coronavirus (SARS-CoV and murine hepatitis virus (MHV)) induce endoplasmic reticulum (ER) stress during infection, as a result of incorrect protein folding or accumulation in the ER lumen [10]. Cells can respond to ER stress by mediating an unfolded-protein response, which requires the transcription of molecular chaperones responsible for protein folding and repression of protein synthesis. Glucose Regulated Protein 78 (GRP78), also known as Binding immunoglobulin protein (BiP), heat shock 70 kDa protein 5 (HSPA5) or Byun1, is a HSP70 molecular chaperone encoded by the HSPA5 gene. This molecular chaperone is usually located in the lumen of the ER but can also be found at the cell surface, cytosol, mitochondria and nucleus. Cell surface relocation of GRP78 is caused by either ER stress (e.g., in some viral infections or some types of cancer) or by GRP78 overexpression [11,12]. It has been described that in stressed cells of respiratory system, GRP78 is overexpressed and translocated from the Endoplasmic Reticulum to the cell membrane [3]. 

Cell surface GRP78 is known to play a role in the infection of host cells by several virus, such as MERS-CoV, Ebola, Dengue, Japanese Encephalitis virus, Coxsackievirus A9 (CAV9) and Borna Disease Virus (BDV) [13]. In addition, four regions of the SARS-CoV-2 Spike protein were predicted by molecular docking to bind the host cell surface GRP78 [14]. Molecular docking prediction suggests that when the virus is approaching the target cell expressing cell-surface GRP78, binding is more favorable between region IV (C480–C488) of the SARS-CoV-2 spike protein and the GRP78 substrate binding domain (SBD) [14]. Even though these molecular docking studies still lack experimental validation in cells, they suggest that inhibiting this interaction could possibly decrease the rate of viral infection. This molecular docking study, together with the abovementioned literature on other viruses, made us hypothesize that GRP78 might be a molecular target to inhibit SARS-CoV-2 infection.

Consequently, inhibitors of this putative interaction might be useful not only to prevent infection but also to contribute to treat this disease. For example, it was recently found that inhibition of the SRC tyrosine kinase blocks cell surface GRP78 [15]. This raises the possibility of repurposing SRC inhibitors and other drugs that are inhibitors of cell surface GRP78 for the treatment of Covid-19. 

Indeed, as there are no specific therapies approved for the treatment of Covid-19, one of the strategies that has been widely used is the repositioning of clinically approved drugs, as they have known safety profiles and are readily available for clinical trials [16,17]. This approach has the advantage of possibly providing fast and safe drug leads. In fact, small-molecules approved for other human diseases, such as immunosuppressants, inhibitors of RNA synthesis and HIV protease inhibitors, amongst others, have reasonable supporting evidence for being considered for further studies regarding treatment of Covid-19 [18]. Curiously, there has been conflicting data on the use of hydroxycloroquine for the management of critically ill patients with Covid-19 [19,20].

In this work, to further suggest that GRP78 is a possible drug target for Covid-19 patients, we performed GRP78 gene expression studies in the blood of SARS-CoV-2 (+) versus SARS-CoV-2 (−) pneumonia patients. Moreover, to identify drugs that are potential inhibitors of GRP78, we performed a structure-based virtual screen using 10,761 small molecules retrieved from DrugBank, using GRP78 nucleotide binding domain (NBD) and SBD as targets.

## 2. Results

### 2.1. GRP78 Gene Expression Studies

GRP78 gene expression studies were conducted in SARS-CoV-2 (−) and SARS-CoV-2 (+) pneumonia patients. The vital parameters of those two groups of patients were also analyzed. The median values and gender distributions were similar in the SARS-CoV-2 (−) and SARS-CoV-2 (+) pneumonia groups (*p* = 0.838 and *p* = 0.928, respectively). The median values of fever, sPO2, CURB-65 Score, pneumonia severity score, systolic and diastolic blood pressure were similar in both groups (*p* = 0.589; *p* = 0.926; *p* = 0.956 and *p* = 0.056) (Table 1).

The GRP78 mRNA levels in the blood were 56.41 ± 1.69 in the SARS-CoV-2 (+) pneumonia group and 14.7 ± 0.95 in the SARS-CoV-2 (−) pneumonia group. Indeed, the GRP78 mRNA level was found statistically significantly higher in SARS-CoV-2 (+) pneumonia group than in SARS-CoV-2 (−) pneumonia group (*p* = 0.0001) (Table 2).

### 2.2. Virtual Screening Studies

**Figure 1:**
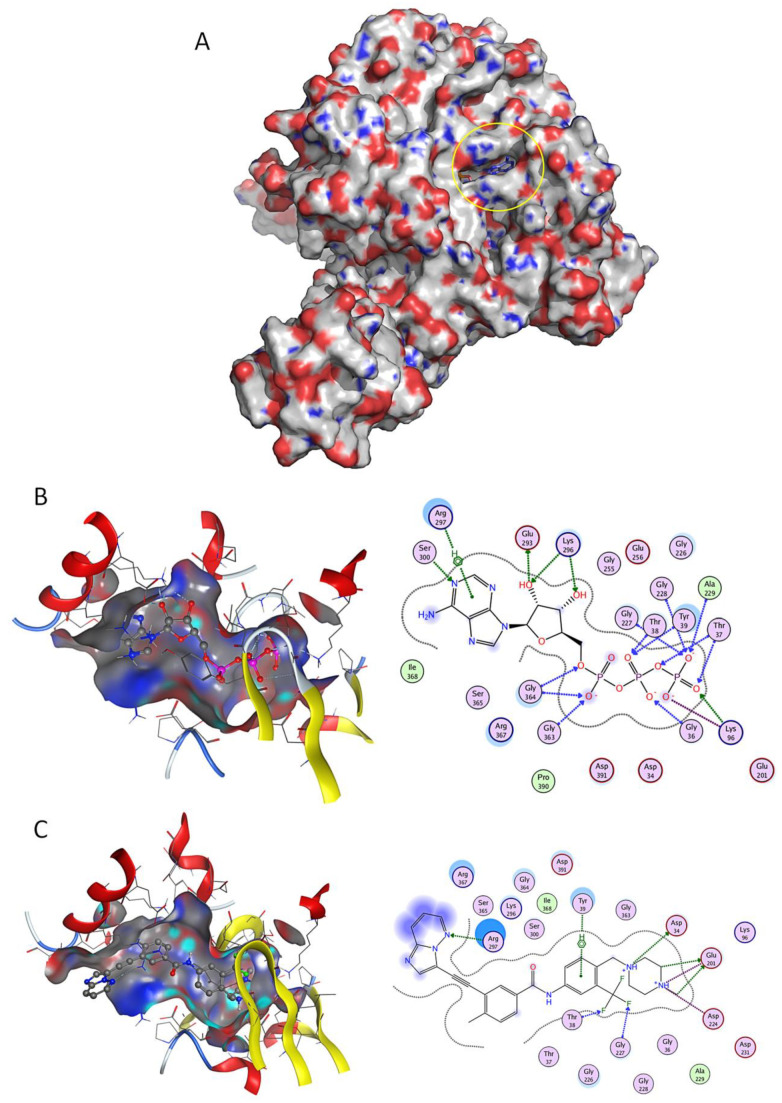
Docking studies on GRP78 nucleotide binding domain (NBD). (**A**) Three-dimensional (3D) surface structure of GRP78 (pdb code 5e84) showing its nucleotide-binding domains (yellow circle). (**B**) Crystallographic ATP, and (**C**) known drug Ponatinib, in 3D representation (left image) and respective two-dimensional (2D) interaction scheme (right image). The polar and non-polar amino-acids are shown in pink and green circles; hydrogen bonding is indicated by dotted arrows; with dotted lines represent arene-hydrogen interactions; proximity contour are dotted lines surrounding the ligand, indicating the shape of the binding site and available space to the more outward-facing parts of the ligand; blue shadows in some amino acids indicate the receptor exposure differences by the size and intensity of the quoits disks. The directions of the shadows indicate the directions of the amino acids toward the ligands. The blue clouds around the ligand atoms indicate the solvent exposure.

When virtually screening the NBD of GRP78, 129 DrugBank molecules presented higher affinity to GRP78 NBD (lower estimated ∆G) than control ATP (Table S1 on Supplementary data and Figure 1). It was possible to validate the docking methodology for NBD, as the obtained docking pose for ATP has superimposed with the co-crystallized ligand structure with an RMSD of 0.752Å.

Imatinib (a BCR-ABL inhibitor) was the top docking score drug (−9.26 kcal/mol) found to bind to NBD (Figure 1A), presenting higher affinity than control ATP (Figure 1B), followed by FK-614 (−8.98 kcal/mol) and Selonsertib (−8.86 kcal/mol) (Table S1). Several other BCR-ABL inhibitors such as Ponatinib (Figure 1C), Nilotinib, Bafetinib, Danusertib and Dasatinib were also retrieved in this structure-based virtual screen. There are several residues involved in ATP binding [21] that were also implicated in the interaction of test ligands and GRP78; for example, residues Thr-38 and Gly-227 established hydrogen interactions with two phosphate groups of ATP (Figure 1B) and also with fluoride atoms of Ponatinib (Figure 1C). 

**Figure 2:**
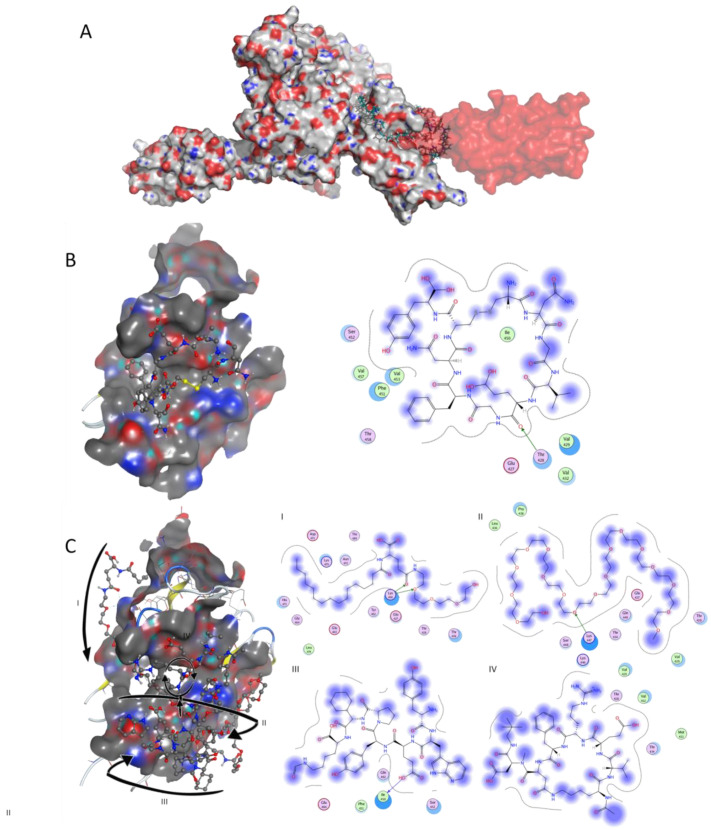
Docking studies on GRP78 SBD. (**A**) 3D surface structure of GRP78 (pdb code 5e84) (grey surface) bound to SARS-CoV-2 (red transparent surface); the C480–C488 section of the whole SARS-CoV-2 spike (pdb code 6m0j) was aligned with the docked C480–C488 in order to allow an easier visualization of the position of the spike towards the GRP78 target. Known peptide Zilucoplan is shown in sticks for exemplification. (**B**) SARS-CoV-2 region IV, and (**C**) known drug Zilucoplan bound to GRP78 SBD in 3D representation (left image, with arrows representing the direction of the peptidic strand from the linear—I, II, III—to the macrocyclic segment—IV) and respective 2D interaction scheme (right image). The polar and non-polar amino-acids are shown in pink and green circles; hydrogen bonding is indicated by dotted arrows; proximity contour are dotted lines surrounding the ligand, indicating the shape of the binding site and available space to the more outward-facing parts of the ligand; blue shadows in some amino acids indicate the receptor exposure differences by the size and intensity of the quoits disks. The directions of the shadows indicate the directions of the amino acids toward the ligands. The blue clouds around the ligand atoms indicate the solvent exposure.

When virtually screening the SBD, results showed that 280 drugbank molecules were predicted to bind more tightly to target GRP78 than SARS-CoV-2 region IV, such as Zilucoplan (−13.53 kcal/mol), Obinepitide (−13.28 kcal/mol) and Corticorelin ovine triflutate (−13.07 kcal/mol) (Table S2 on Supplementary Data). It has recently been predicted that the region IV (C480–C488) of the SARS-CoV-2 spike protein binds to GRP78 SBD [14] (Figure 2A). The surface-accessible region IV of the spike protrudes to the outer side of the virus facing the target cell and establishing hydrophobic interactions and an hydrogen bond (Figure 2B). Zilucoplan (Figure 2C) was predicted to be suitable to bind the cell-surface GRP78, as it is a long molecule with several aromatic groups, expanding throughout the target surface and establishing several hydrogen interactions (Figure 2C). 

## 3. Discussion

The SARS-CoV-2 Spike protein was recently predicted by molecular docking to bind the host cell surface GRP78 [14]. Thus, GRP78 was suggested to function as a receptor for SARS-CoV-2. In this work, we verified that GRP78 gene expression was increased in the blood of SARS-CoV-2 (+) versus SARS-CoV-2 (−) pneumonia patients. This is in agreement with some results previously obtained by some of us, which showed that higher serum GRP78 protein concentrations were found in the SARS-CoV-2 infected patients when compared to patients with pneumonia or controls [22]. GRP78 protein is a member of the HSP-70 protein family. It is known that, during Acute Respiratory Syndrome Distress (ARDS), endothelial barrier dysfunction occurs in the lung tissue, the production of heat shock proteins occurs and their release into the blood increases [23,24]. Indeed, it has been demonstrated that HSP-70 proteins are released into the blood in severe traumas. Additionally, it was revealed that HSP-70 protein levels were higher in ARDS cases [25]. Therefore, it is possible that the high levels of GRP78 found in the blood of SARS-CoV-2 (+) patients could be derived from the lung tissue. Further work will be necessary to confirm this hypothesis. Interestingly, another study recently showed confirmatory proof for the presence of GRP78 protein in vitro in airway epithelial cells, further allowing to hypothesize that GRP78 may be a receptor for SARS-CoV-2, facilitating initial host cell infection [26]. Therefore, our results, together with the ones published by the abovementioned studies, allow to hypothesize that GRP78 may be a receptor for SARS-CoV-2, and therefore, a good molecular target to treat Covid-19.

Remarkably, inhibiting GRP78 limited Dengue Virus infection [27]. Moreover, reduction of GRP78 levels by small interfering RNAs blocked the entry of Japanese Encephalitis Virus into cells [28]. Furthermore, decreasing GRP78 expression with siRNAs inhibited Ebola virus replication and pre-treatment with the small molecule (-)-epigallocatechin gallate inhibited Ebola virus infection [29].

Therefore, we aimed to identify drugs that could be repurposed to inhibit GRP78, which thus have potential anti-SARS-CoV-2 activity by possibly stopping or preventing SARS-CoV-2 infection. The DrugBank database is a comprehensive, freely accessible, online database containing information on drugs and drug targets [30], which can be a valuable source for the search of known drugs/bioactive compounds with off-target anti-Covid-19 activity. This database was selected over other known databases because it has links to almost all major biochemoinformatics and drug/pharmaceutical databases [31], allowing an easy localization and acquisition of the desired molecules for further testing. 

The inhibition of the interaction between the SARS-CoV-2 spike protein and the host cell receptor by blocking GRP78 SBD is an interesting strategy to possibly identify drugs that decrease the rate of viral infection [14]. Another potential mechanism of inhibition of SARS-CoV-2 infection might be the inhibition of ATPase activity by binding to the GRP78 nucleotide binding domain (NBD) [32]. Therefore, a structure-based virtual screen was performed using 10,761 small molecules retrieved from DrugBank, using GRP78 NBD and SBD as targets, and using ATP and SARS-CoV-2 peptide C480–C488 as controls, respectively.

Interestingly, the BCR-ABL inhibitor Imatinib was the top docking score drug found in this virtual screening on GRP78 NBD. In agreement with this, a high-throughput screen of FDA-approved drugs identified Imatinib as an inhibitor of both SARS-CoV and MERS-CoV [33]. In addition, this study showed that the anti-SARS-CoV activity of Imatinib occurred at the early stages of infection, after internalization and endosomal trafficking, by inhibiting fusion of the virions at the endosomal membrane. This study also showed that Abelson tyrosine-protein kinase 2 (ABL2) was found necessary for efficient SARS-CoV and MERS-CoV replication in vitro. Most interestingly, a randomized non-comparative phase 2 pilot clinical trial will start soon to test the value of Imatinib mesylate as an early treatment of Covid-19 disease in aged hospitalized patients. The description of this clinical trial states that “The EC_50_ of imatinib for the inhibition of the virus is under investigation but we now have a first estimates with EC_50_ close to 2.5 microM. This plasmatic concentration is achievable with imatinib 800 mg/dL” [34].

Several other BCR-ABL inhibitors such as Ponatinib, Nilotinib, Flumatinib, Bafetinib, Danusertib and Dasatinib were also retrieved in this structure-based virtual screen. In addition, other BCR-ABL inhibitors (not listed in Table S1 due to docking scores higher than ATP) such as GNF-2, Tozasertib, Revastinib, or Bosutinib, presented highly negative free energy of binding, lower than some of the known GRP78 NBD inhibitors such as honokiol [35], hkh40a [36], VER-155008 [37], isoliquiritigenin [38] or epigallocatechin gallate [32], suggesting that in general this class of compounds is worth testing for further understanding the molecular mechanism of GRP78 modulation. 

Several other protein kinase inhibitors were identified, such as Sorafenib (VEGFR, PDGFR and RAF inhibitor) [39], Dacomitinib (EGFR inhibitor) [40], Neratinib (HER2 inhibitor) [41], Ponatinib (FGFR, PDGFR, SRC, RET, KIT and FLT1 inhibitor) [42], Gedatolisib (PI3K/mTOR inhibitor) [43], Danusertib (aurora kinase inhibitor) [44], Glesatinib (c-Met inhibitor) [45], Tivozanib (VEGFR inhibitor) [46], Pyrotinib (HER2 and EGFR inhibitor) [47] or Olaparib (PARP inhibitor) [48], among others.

Interestingly, two of these drugs are inhibitors of SRC (Bosutinib and Ponatinib) and were previously patented as also being capable of blocking cell surface GRP78 expression [15].

As there is no available crystallographic complex of GRP78-SARS-CoV-2, the validation of the docking onto SBD was achieved by comparison with available data in the literature (SARS-CoV-2 residues 486–788 are facing GRP78 Phe-451 and Ser-452, while SARS-CoV-2 residues 483–484 are facing Thr-428) [14]. The SARS-CoV-2 C480-C488 loop has a rigid and well defined structure that is preserved along molecular dynamics simulations, which allows the establishment of several stable interactions with targets such as ACE-II [49]. The SARS-CoV-2 segment C480–C488 was sectioned from the spike structure (pdb code 6m0j) and the docking protocol used was the same that was applied to the other ligands: it was treated as flexible and it was kept as a cyclic peptide during the docking simulation (due to a disulphide bond between Cys-480 and Cys-488) [50]. In fact, the cyclic form has already been described for other peptides, such as Pep42, as being determinant for GRP78 recognition due to the stabilization of hydrophobic interactions by the rigid cyclic structure [13]. Interestingly, Zilucoplan, Obinepitide and Corticorelin ovine triflutate were found to have lower free energy of binding (higher affinity) to GRP78 SBD than SARS-CoV-2 (C480–C488), which suggests that these compounds may compete with the virus spike for binding, thus preventing the internalization of SARS-CoV-2 and consequent infection. Interestingly, the compound with the highest binding affinity docked onto SBD was Zilucoplan, a macrocyclic peptide, such as SARS-CoV-2 C480-C488 loop [14] and known GRP78 inhibitor Pep42 [13]

GRP78 has high affinity for hydrophobic or hydrophilic regions of protein substrates such as that of the SARS-CoV-2 spike [14]. Hence, compounds with high affinity to this GRP78 binding pocket could theoretically compete with the virus spike for binding [51]. As GRP78 may act as a co-receptor for virus internalization by association with other molecules on the cell surface, as previously reported for other virus [52,53], the compounds retrieved on the virtual screening may be potential inhibitors of internalization of SARS-CoV-2, thus preventing infection. Interestingly, a recent study showed by molecular docking that some natural products (such as phytoestrogens and estrogens) also seem to bind SBD GRP78. The author of this study suggested that these natural products may interfere with SARS-CoV-2 attachment to stressed cells [51].

Concerning the NBD, simulation studies have previously reported that after binding of a known competitive inhibition to GRP78 ATP-binding site, the SBD adopts a conformation that has low affinity for substrates, thus blocking transportation [54]. In fact, the binding of several inhibitors to the ATP-binding site has already been revealed by several crystallographic studies [55]. Therefore, the tested small molecules may block GRP78 ATPase activity by directly binding to the ATP-binding site, thus impairing GRP78 function and theoretically avoiding SARS-CoV-2 infection. 

Further in vitro testing will be performed in a near future to validate the in silico findings herein presented. This will be relevant not only to identify drugs to possibly treat Covid-19 but also other diseases in which GRP78 has been acknowledge as a good molecular target, such as some cancers (particularly chemoresistant cancers), and atherosclerotic, thrombotic and auto-immune diseases [11,56,57].

## 4. Materials and Methods 

### 4.1. Gene Expression Studies

#### 4.1.1. Patients 

A total of 34 patients (10 patients diagnosed with pneumonia but SARS-CoV-2 negative and 24 patients diagnosed with pneumonia and confirmed with SARS-COV-2 infection) who were admitted to the emergency department of the Pamukkale University Hospital with symptoms of pneumonia and SARS-COV-2 infection were included in the study. All SARS-CoV-2 positive patients were at an early phase of the disease. Prior to the study, ethical approval numbered 60116787-020/26598 was obtained from Pamukkale University Ethics Committee. All the procedures were in compliance with the Helsinki Declaration. All the patients were informed about the study and a written consent form was obtained from all patients wishing to participate in the study. Researchers analyzed only deidentified (anonymized) data.

The measured vital parameters (body temperature, sPO2, and blood pressure) of the patients were recorded in the emergency service. Pneumonia severity index (PSI Score) and CURB-65 scores of patients were calculated as known in the literature. PSI score and CURB-65 score were used to determine the clinical status of the patients. 

#### 4.1.2. Gene Expression Analysis

Gene expression analysis of glucose regulating protein 78 (GRP78) was analyzed in 10 patients with pneumonia and 24 patients with SARS-COV-2 infection. Blood samples were collected from individuals to PaxGene tubes. RNA was extracted with Human Blood RNA Purification Kit (GMBiolabs, Taichung City, Taiwan) according to the procedure recommended by manufacturer. The mRNA level was studied from the blood samples taken when the patients applied to the emergency room.

Conversion of total RNA to single-strand complementary DNA (cDNA) was done with High-Capacity cDNA Reverse Transcription Kit (Applied Biosystems Inc., Foster City, CA, USA) with random primers. PCR reactions were performed with the Taq^®^Man Universal PCR Master Mix (15 μL, Applied Biosystems, individual Taq^®^Man Gene Expression Assays) by using 5 μL of diluted cDNA, 1 μL 200 nmol/L of the labeled probe, and 1.5 μL pre-developed primer-probe sets for GRP78 (Applied Biosystems assay ID Hs99999174_m1) was used [58]. Based on the CT value and the corresponding standard curve, the mRNA quantity of each sample was calculated by determining the ratio between the amounts of the gene of interest and GAPDH. Primers and probe sequences for GAPDH are available from the authors on request.

#### 4.1.3. Statistical Analysis

Data were analyzed using SPSS 17 (IBM Corp., SPSS Inc., Chicago IL, USA) statistical software package. While mean values, standard deviations and medians (IQR) were provided as continuous variables, categorical variables were presented in number and percentage. Mann-Whitney U-test was used to compare the independent group differences when the parametric test assumptions were not met. Student’s *t* test was used to compare the independent group differences when the parametric test assumptions were met. Differences between categorical variables were analyzed by Chi-square analysis. A *p*-value < 0.05 was considered statistically significant in all analyses.

### 4.2. Molecular Docking Virtual Screening

#### 4.2.1. Preparation of Receptor and Ligands

The 3D structure of GRP78 was obtained from the Protein Data Bank (PDB code: 5e84) at 2.99 Å resolution [59] and loaded to Molecular Operating Environment (MOE^®^) version 2014 from Chemical Computing Group (CCG) [60]. The preparation of the target protein involved the following steps: removal of all water molecules and ATP; addition of hydrogen atoms; calculation of atomic partial charges according to the Amber12:EHT force field. 10,761 small molecule drugs 3D structures were obtained from the DrugBank database (Version 5.1.5) [30]. The molecule wash function was used to generate meaningful protonation states by deprotonating strong acids and protonating strong bases. Energy minimization of all molecules was performed using the Amber12:EHT force field at a RMSD gradient of 0.01 kcal/mol.Å. The existing chirality was preserved and partial charges were calculated according to the standard parameters of the force field. 

#### 4.2.2. Molecular Docking

The docking of DrugBank database molecules into the nucleotide-binding domain (NBD) and the SARS-CoV2 binding domain (SBD) of GRF78 was performed using MOE-Dock implemented on MOE. The default placement method, triangle matcher algorithm, was selected for pose generation by aligning the ligand triplets on the alpha sphere triplets of the receptor. Two rescoring functions, including London dG and GBVI/WSA dG, were utilized for pose scoring. Results were ranked according to their S score (kcal/mol).

## 5. Conclusions

In spite of all the efforts that have been made worldwide, there is still no drug available to effectively treat Covid-19. SARS-CoV-2 mainly affects the host by binding to membrane receptors through its spike glycoprotein. One of the suggested putative receptors was GRP78. Here, we showed that GRP78 gene expression in blood was higher in SARS-CoV-2 (+) versus SARS-CoV-2 (−) pneumonia patients, providing further indication that GRP78 might be a good molecular target to treat Covid-19. Therefore, interfering with this interaction could theoretically be a possible approach to avoid the viral infection and propagation. We suggest that further work should be urgently carried out to confirm if GRP78 is indeed a good molecular target to treat Covid-19.

Virtual screening tools can be used to rapidly identify known drugs that could be repurposed to act as GRP78 inhibitors, and thus, theoretically have potential as anti-SARS-CoV-2 drugs. Indeed, our in silico data identified several GRP78 inhibitors, which may be good candidates for anti-SARS-CoV-2 activity. A total of 409 compounds were identified with potential to block the theoretical binding of the viral Spike protein with the host GRP78, which therefore have potential to stop or prevent infection. The most promising identified drugs that could be repurposed were: Imatinib, FK-614 and Selonsertib (GRP78 NBD binders); Zilucoplan, Obinepitide and Corticorelin ovine triflutate (GRP78 SBD binders). Since these preliminary results were only based on in silico work, future in vitro testing is necessary to confirm the antiviral activity of these compounds against SARS-CoV-2.

## Figures and Tables

**Table 1:** Vital and clinical parameters of the groups.

Parameters	SARS-CoV-2 (-) Pneumonia (*n* = 24) Median (IQR)	SARS-CoV-2 (+) Pneumonia (*n* = 10) Median (IQR)	*p*-Value
**Gender Male**	14 (41.17%)	6 (60%)	*p*^1^ = 0.928
**Female**	10 (58.83%)	4 (40%)	
**Age**	52.5 (37–75.75)	60.5 (36.5–72.5)	*p*^2^ = 0.838
**Fever (°C)**	36.85 (36.525–37.675)	36.7 (36.27–37.35)	*p*^2^ = 0.589
**sPO_2_**	95 (92.25–97)	95 (93–97.25)	*p*^2^ = 0.926
**Sys. BP (mm/Hg)**	127 (110–140.75)	128 (110–142.5)	*p*^2^ = 0.956
**Dias. BP (mm/Hg)**	80 (70.5–88.75)	70 (61.5–80)	*p*^2^ = 0.056
**PSI Score**	76.5 (38.25–113)	82.5 (51–118)	*p*^2^ = 0.642
**CURB-65 Score**	1 (0.5–2)	1 (1–2)	*p*^2^ = 0.341

°C Celsius degree; sPO_2_, partial oxygen saturation; Sys. BP, systolic blood pressure; Dias. BP, diastolic blood pressure. *p*^1^ value is derived from fisher exact test. *p*^2^ values are derived from Mann Whitney U test. PSI; Pneumonia severity Index.

**Table 2:** GRP78 gene expression levels in the blood of SARS-CoV-2 (−) and SARS-CoV-2 (+) pneumonia patients.

Patient Groups	SARS-CoV-2(−) Pneumonia (*n* = 24)		SARS-CoV-2(+) Pneumonia (*n* = 10)		*p*-Value
	Mean ± SD	Median (IQR)	Mean ± SD	Median (IQR)	
**GRP-78 mRNA Levels**	14.7 ± 0.95	14.7 (14.26–15.25)	56.41 ± 1.69	57.09 (54.09–57.69)	* 0.0001

*p*-Value is derived from Student’s *t* test and it shows comparison between the groups. GRP-78, Glucose regulated protein-78; SD, standard deviation. * 0.0001 means there is a statistically difference between the groups.

## Supplementary Materials

The following are available online at https://www.mdpi.com/1424-8247/13/6/132/s1, Table S1: Results of the docking of DrugBank compounds onto GRP78 (NBD) (only molecules with lower docking scores than control ATP are presented). Table S2: Results of the docking of DrugBank compounds onto GRP78 (SBD) (only molecules with lower docking scores than control region IV (C480-C488) from SARS-CoV-2 spike are presented).

## References

[1] Schoeman D., Fielding B.C. (2019). Coronavirus envelope protein: Current knowledge. Virol. J..

[2] Coronaviridae Study Group of the International Committee on Taxonomy of Viruses (2020). The species Severe acute respiratory syndrome-related coronavirus: Classifying 2019-nCoV and naming it SARS-CoV-2. Nat. Microbiol..

[3] Walls A.C., Park Y.-J., Tortorici M.A., Wall A., McGuire A.T., Veesler D. (2020). Structure, Function, and Antigenicity of the SARS-CoV-2 Spike Glycoprotein. Cell.

[4] Ou X., Liu Y., Lei X., Li P., Mi D., Ren L., Guo L., Guo R., Chen T., Hu J. (2020). Characterization of spike glycoprotein of SARS-CoV-2 on virus entry and its immune cross-reactivity with SARS-CoV. Nat. Commun..

[5] Shang J., Ye G., Shi K., Wan Y., Luo C., Aihara H., Geng Q., Auerbach A., Li F. (2020). Structural basis of receptor recognition by SARS-CoV-2. Nature.

[6] Wan Y., Shang J., Graham R., Baric R.S., Li F. (2020). Receptor Recognition by the Novel Coronavirus from Wuhan: An Analysis Based on Decade-Long Structural Studies of SARS Coronavirus. J. Virol..

[7] Zhou P., Yang X.-L., Wang X.-G., Hu B., Zhang L., Zhang W., Si H.-R., Zhu Y., Li B., Huang C.-L. (2020). A pneumonia outbreak associated with a new coronavirus of probable bat origin. Nature.

[8] Nadeem M.S., Zamzami M.A., Choudhry H., Murtaza B.N., Kazmi I., Ahmad H., Shakoori A. (2020). Origin, Potential Therapeutic Targets and Treatment for Coronavirus Disease (COVID-19). Pathogens.

[9] Shereen M.A., Khan S., Kazmi A., Bashir N., Siddique R. (2020). COVID-19 infection: Origin, transmission, and characteristics of human coronaviruses. J. Adv. Res..

[10] Versteeg G.A., van de Nes P.S., Bredenbeek P.J., Spaan W.J. (2007). The Coronavirus Spike Protein Induces Endoplasmic Reticulum Stress and Upregulation of Intracellular Chemokine mRNA Concentrations. J. Virol..

[11] Quinones Q.J., de Ridder G., Pizzo S.V. (2008). GRP78: A chaperone with diverse roles beyond the endoplasmic reticulum. Histol. Histopathol..

[12] Zhang Y., Liu R., Ni M., Gill P., Lee A.S. (2010). Cell Surface Relocalization of the Endoplasmic Reticulum Chaperone and Unfolded Protein Response Regulator GRP78/BiP. J. Boil. Chem..

[13] Ibrahim I.M., Abdelmalek D.H., ElFiky A.A. (2019). GRP78: A cell’s response to stress. Life Sci..

[14] Ibrahim I.M., Abdelmalek D.H., Elshahat M.E., ElFiky A.A. (2020). COVID-19 spike-host cell receptor GRP78 binding site prediction. J. Infect..

[15] Lee A.S., Tsai Y. Patent Application Publication: SRC inhibitor to block cell surface GRP78 expression 2019. http://www.freepatentsonline.com/y2019/0076431.html.

[16] Rosa S.G.V., Santos W.C. (2020). Clinical trials on drug repositioning for COVID-19 treatment. Rev. Panam. Salud Pública.

[17] Serafin M.B., Bottega A., Foletto V.S., da Rosa T.F., Hörner A., Hörner R. (2020). Drug repositioning is an alternative for the treatment of coronavirus COVID-19. Int. J. Antimicrob. Agents.

[18] Guy R.K., di Paola R.S., Romanelli F., Dutch R.E. (2020). Rapid repurposing of drugs for COVID-19. Science.

[19] Huang M., Tang T., Pang P., Li M., Ma R., Lu J., Shu J., You Y., Chen B., Liang J. (2020). Treating COVID-19 with Chloroquine. J. Mol. Cell Boil..

[20] Mehra M.R., Ruschitzka F., Patel A.N. (2020). Retraction—Hydroxychloroquine or chloroquine with or without a macrolide for treatment of COVID-19: A multinational registry analysis. Lancet.

[21] Hughes S.J., Antoshchenko T., Chen Y., Lü H., Pizarro J.C., Park H.-W. (2016). Probing the ATP Site of GRP78 with Nucleotide Triphosphate Analogs. PLoS ONE.

[22] Koseler A., Sabirli R., Goren T., Turkçuer I., Kurt O. (2020). Endoplasmic Reticulum Stress Markers in SARS-COV-2 Infection and Pneumonia: Case-Control Study. In Vivo.

[23] Barabutis N. (2019). Unfolded Protein Response in Acute Respiratory Distress Syndrome. Lung.

[24] Bromberg Z., Deutschman C.S., Weiss Y.G. (2005). Heat shock protein 70 and the acute respiratory distress syndrome. J. Anesthesia.

[25] Haider T., Simader E., Glück O., Ankersmit H.J., Heinz T., Hajdu S., Negrin L.L. (2019). Systemic release of heat-shock protein 27 and 70 following severe trauma. Sci. Rep..

[26] Aguiar J.A., Tremblay B.J.-M., Mansfield M.J., Woody O., Lobb B., Banerjee A., Chandiramohan A., Tiessen N., Dvorkin-Gheva A., Revill S. (2020). Gene Expression and in Situ Protein Profiling of Candidate SARS-CoV-2 Receptors in Human Airway Epithelial Cells and Lung Tissue.

[27] Chen H.-H., Chen C.-C., Lin Y.-S., Chang P.-C., Lu Z.-Y., Lin C.-F., Chen C.-L., Chang C.-P. (2017). AR-12 suppresses dengue virus replication by down-regulation of PI3K/AKT and GRP78. Antivir. Res..

[28] Nain M., Mukherjee S., Karmakar S.P., Paton A.W., Paton J.C., Abdin M.Z., Basu A., Kalia M., Vrati S. (2017). GRP78 Is an Important Host Factor for Japanese Encephalitis Virus Entry and Replication in Mammalian Cells. J. Virol..

[29] Reid S.P., Shurtleff A.C., Costantino J.A., Tritsch S.R., Retterer C., Spurgers K.B., Bavari S. (2014). HSPA5 is an essential host factor for Ebola virus infection. Antivir. Res..

[30] Wishart D.S., Feunang Y.D., Guo A.C., Lo E.J., Marcu A., Grant J.R., Sajed T., Johnson D., Li C., Sayeeda Z. (2017). DrugBank 5.0: A major update to the DrugBank database for 2018. Nucleic Acids Res..

[31] Wishart D.S., Knox C., Guo A.C., Cheng D., Shrivastava S., Tzur D., Gautam B., Hassanali M. (2007). DrugBank: A knowledgebase for drugs, drug actions and drug targets. Nucleic Acids Res..

[32] Ermakova S.P., Kang B.S., Choi B.Y., Schuster T.F., Ma W.-Y., Bode A.M., Dong Z. (2006). Epigallocatechin Gallate Overcomes Resistance to Etoposide-Induced Cell Death by Targeting the Molecular Chaperone Glucose-Regulated Protein 78. Cancer Res..

[33] Dyall J., Coleman C., Hart B., Venkataraman T., Holbrook M.R., Kindrachuk J., Johnson R.F., Olinger G., Jahrling P.B., Laidlaw M. (2014). Repurposing of Clinically Developed Drugs for Treatment of Middle East Respiratory Syndrome Coronavirus Infection. Antimicrob. Agents Chemother..

[34] Versailles-Hospital (2020). A Randomized Non-Comparative Phase 2 Pilot Study Testing the Value of Imatinib Mesylate as an Early Treatment of Covid-19 Disease in Aged Hospitalized Patients; NCT04357613, France. NCT04357613.

[35] Martin S., Lamb H.K., Brady C., Lefkove B., Bonner M.Y., Thompson P., E Lovat P., Arbiser J.L., Hawkins A.R., Redfern C.P. (2013). Inducing apoptosis of cancer cells using small-molecule plant compounds that bind to GRP78. Br. J. Cancer.

[36] Kosakowska-Cholody T., Lin J., Srideshikan S.M., Scheffer L., I Tarasova N., Acharya J. (2014). HKH40A downregulates GRP78/BiP expression in cancer cells. Cell Death Dis..

[37] Asling J., Morrison J., Mutsaers A. (2016). Targeting HSP70 and GRP78 in canine osteosarcoma cells in combination with doxorubicin chemotherapy. Cell Stress Chaperon.

[38] Wang N., Wang Z., Peng C., You J., Shen J., Han S., Chen J. (2014). Dietary compound isoliquiritigenin targets GRP78 to chemosensitize breast cancer stem cells via β-catenin/ABCG2 signaling. Carcinogenesis.

[39] Wilhelm S.M., Adnane L., Newell P., Villanueva A., Llovet J.M., Lynch M. (2008). Preclinical overview of sorafenib, a multikinase inhibitor that targets both Raf and VEGF and PDGF receptor tyrosine kinase signaling. Mol. Cancer Ther..

[40] Nagano T., Tachihara M., Nishimura Y. (2019). Dacomitinib, a second-generation irreversible epidermal growth factor receptor tyrosine kinase inhibitor (EGFR-TKI) to treat non-small cell lung cancer. Drugs Today.

[41] Paranjpe R., Basatneh D., Tao G., de Angelis C., Noormohammed S., Ekinci E., Abughosh S., Ghose R., Trivedi M.V. (2019). Neratinib in HER2-Positive Breast Cancer Patients. Ann. Pharmacother..

[42] Tan F.H., Putoczki T.L., Stylli S.S., Luwor R. (2019). Ponatinib: A novel multi-tyrosine kinase inhibitor against human malignancies. OncoTargets Ther..

[43] Langdon S.P., Kay C., Um I.H., Dodds M., Muir M., Sellar G., Kan J., Gourley C., Harrison D.J. (2019). Evaluation of the dual mTOR/PI3K inhibitors Gedatolisib (PF-05212384) and PF-04691502 against ovarian cancer xenograft models. Sci. Rep..

[44] Meulenbeld H., Mathijssen R.H., Verweij J., de Wit R., de Jonge M.J. (2012). Danusertib, an aurora kinase inhibitor. Expert Opin. Investig. Drugs.

[45] Engstrom L.D., Aranda R., Lee M., Tovar E.A., Essenburg C., Madaj Z., Chiang H., Briere D., Hallin J., Lopez-Casas P.P. (2017). Glesatinib Exhibits Antitumor Activity in Lung Cancer Models and Patients HarboringMETExon 14 Mutations and Overcomes Mutation-mediated Resistance to Type I MET Inhibitors in Nonclinical Models. Clin. Cancer Res..

[46] De Luca A., Normanno N. (2010). Tivozanib, a pan-VEGFR tyrosine kinase inhibitor for the potential treatment of solid tumors. IDrugs Investig. Drugs J..

[47] Huang L.-T., Ma J.-T., Zhang S.-L., Li X.-H., Sun L., Jing W., Zhao J.-Z., Wang Y.-R., Han C.-B. (2019). Durable Clinical Response to Pyrotinib After Resistance to Prior Anti-HER2 Therapy for HER2-Positive Advanced Gastric Cancer: A Case Report. Front. Oncol..

[48] Verhagen C.V., de Haan R., Hageman F., Oostendorp T.P., Carli A.L., O’Connor M.J., Jonkers J., Verheij M., Brekel M.W.V.D., Vens C. (2015). Extent of radiosensitization by the PARP inhibitor olaparib depends on its dose, the radiation dose and the integrity of the homologous recombination pathway of tumor cells. Radiother. Oncol..

[49] Spinello A., Saltalamacchia A., Magistrato A. (2020). Is the Rigidity of SARS-CoV-2 Spike Receptor-Binding Motif the Hallmark for Its Enhanced Infectivity? Insights from All-Atom Simulations. J. Phys. Chem. Lett..

[50] Hati S., Bhattacharyay S. (2020). Impact of Thiol-Disulfide Balance on the Binding of Covid-19 Spike Protein with Angiotensin Converting Enzyme 2 Receptor.

[51] Elfiky A.A. (2020). Natural products may interfere with SARS-CoV-2 attachment to the host cell. J. Biomol. Struct. Dyn..

[52] Triantafilou K., Fradelizi D., Wilson K., Triantafilou M. (2002). GRP78, a Coreceptor for Coxsackievirus A9, Interacts with Major Histocompatibility Complex Class I Molecules Which Mediate Virus Internalization. J. Virol..

[53] Wu Y.-P., Chang C.-M., Hung C.-Y., Tsai M.-C., Schuyler S.C., Wang R. (2011). Japanese encephalitis virus co-opts the ER-stress response protein GRP78 for viral infectivity. Virol. J..

[54] Gurusinghe K.R.D.S.N.S., Mishra A., Mishra S. (2018). Glucose-regulated protein 78 substrate-binding domain alters its conformation upon EGCG inhibitor binding to nucleotide-binding domain: Molecular dynamics studies. Sci. Rep..

[55] Macias A., Williamson D.S., Allen N., Borgognoni J., Clay A., Daniels Z., Dokurno P., Drysdale M.J., Francis G.L., Graham C.J. (2011). Adenosine-Derived Inhibitors of 78 kDa Glucose Regulated Protein (Grp78) ATPase: Insights into Isoform Selectivity. J. Med. Chem..

[56] Birukova A.A., Singleton P.A., Gawlak G., Tian X., Mirzapoiazova T., Mambetsariev B., Dubrovskyi O., Oskolkova O., Bochkov V.N., Birukov K.G. (2014). GRP78 is a novel receptor initiating a vascular barrier protective response to oxidized phospholipids. Mol. Boil. Cell.

[57] Girona J., Rodríguez-Borjabad C., Ibarretxe D., Vallvé J.-C., Ferré R., Heras M., Rodríguez-Calvo R., Guaita-Esteruelas S., Martínez-Micaelo N., Plana N. (2019). The Circulating GRP78/BiP Is a Marker of Metabolic Diseases and Atherosclerosis: Bringing Endoplasmic Reticulum Stress into the Clinical Scenario. J. Clin. Med..

[58] Langer R., Feith M., Siewert J., Wester H.-J., Hoefler H. (2008). Expression and clinical significance of Glucose Regulated Proteins GRP78 (BiP) and GRP94 (GP96) in human adenocarcinomas of the esophagus. BMC Cancer.

[59] Yang J., Nune M., Zong Y., Zhou L., Liu Q. (2015). Close and Allosteric Opening of the Polypeptide-Binding Site in a Human Hsp70 Chaperone BiP. Structure.

[60] Vilar S., Cozza G., Moro S. (2008). Medicinal chemistry, and the molecular operating environment (MOE): Application of QSAR and molecular docking to drug discovery. Curr. Top. Med. Chem..

