# Correction: Palmeira et al. Preliminary Virtual Screening Studies to Identify GRP78 Inhibitors Which May Interfere with SARS-CoV-2 Infection. *Pharmaceuticals* 2020, *13*, 132

**DOI:** 10.3390/ph17040411

**Published:** 2024-03-25

**Authors:** Andreia Palmeira, Emília Sousa, Aylin Köseler, Ramazan Sabirli, Tarık Gören, İbrahim Türkçüer, Özgür Kurt, Madalena M. Pinto, M. Helena Vasconcelos

**Affiliations:** 1 Laboratory of Organic and Pharmaceutical Chemistry (LQOF), Department of Chemical Sciences, Faculty of Pharmacy, University of Porto, Rua de Jorge Viterbo Ferreira, 228, 4050-313 Porto, Portugal; andreiapalmeira@gmail.com (A.P.); esousa@ff.up.pt (E.S.); madalenakijjoa@gmail.com (M.M.P.); 2 Interdisciplinary Centre of Marine and Environmental Research (CIIMAR), 4450-208 Matosinhos, Portugal; 3 Department of Biophysics, Pamukkale University Faculty of Medicine, 20190 Denizli, Turkey; 4 Department of Emergency Medicine, Kafkas University Faculty of Medicine, 36000 Kars, Turkey; ramazan_sabirli@hotmail.com; 5 Department of Emergency Medicine, Pamukkale University Faculty of Medicine, 20190 Denizli, Turkey; tarikgoren92@hotmail.com (T.G.); iturkcuer@pau.edu.tr (İ.T.); 6 Department of Microbiology, Acibadem Mehmet Ali Aydinlar University School of Medicine, 34752 Istanbul, Turkey; ozgur.kurt@acibadem.edu.tr; 7 Instituto de Investigação e Inovação em Saúde (i3S), University of Porto, 4200-135 Porto, Portugal; 8 Cancer Drug Resistance Group, Institute of Molecular Pathology and Immunology of the University of Porto (IPATIMUP), 4200-135 Porto, Portugal; 9 Laboratory of Microbiology, Department of Biological Sciences, Faculty of Pharmacy, University of Porto, 4050-313 Porto, Portugal

## Text Correction

There was an error in the original publication. In the introduction of the paper [1], GRP78 was incorrectly also named as Byun1.

A correction has been made to Introduction, third paragraph:

Glucose Regulated Protein 78 (GRP78), also known as Binding immunoglobulin protein (BiP) or heat shock 70 kDa protein 5 (HSPA5), is a HSP70 molecular chaperone encoded by the HSPA5 gene. 

The authors state that the scientific conclusions are unaffected. This correction was approved by the Academic Editor. The original publication has also been updated.

## References

[1] Palmeira A., Sousa E., Köseler A., Sabirli R., Gören T., Türkçüer İ., Kurt Ö., Pinto M.M., Vasconcelos M.H. (2020). Preliminary Virtual Screening Studies to Identify GRP78 Inhibitors Which May Interfere with SARS-CoV-2 Infection. Pharmaceuticals.

