# Supplementary material for: Preliminary Virtual Screening Studies to Identify GRP78 Inhibitors Which May Interfere with SARS-CoV-2 Infection
Source: Pharmaceuticals (Basel). 2020 Jun 25;13(6):132. doi: 10.3390/ph13060132 (PMC7345920; doi:10.3390/ph13060132)
Supplement: Supplementary file 1 [file pharmaceuticals-13-00132-s001.pdf]

## Supplementary data

**Table S1.** Results of the docking of DrugBank compounds onto GRP78 (NBD) (only molecules with lower docking scores than control ATP are presented).

| **Molecule name** | **Final docking score S (kcal/mol)** |
|---|---|
| Imatinib | -9.26206 |
| FK-614 | -8.9803 |
| Selonsertib | -8.85865 |
| Sorafenib | -8.841712 |
| CID 5288250 | -8.6442 |
| Pemetrexed | -8.6247129 |
| 4SC-203 | -8.61179 |
| Zafirlukast | -8.59865 |
| (2S)-2-[[4-[2-[(6S)-2-Amino-4-oxo-5,6,7,8-tetrahydro-3H-pyrido[2,3-d]pyrimidin-6-yl]ethyl]benzoyl]amino]pentanedioic acid | -8.58657 |
| Icariin | -8.46474 |
| Raltegravir | -8.44994 |
| 4-[(5-{[4-(3-Chlorophenyl)-3-oxopiperazin-1-YL]methyl}-1H-imidazol-1-YL)methyl]benzonitrile | -8.44899 |
| Dacomitinib | -8.4483 |
| [(1S)-1-Cyclohexyloxycarbonyloxyethyl] 2-ethoxy-3-[[4-[2-(2H-tetrazol-5-yl)phenyl]phenyl]methyl]benzimidazole-4-carboxylate | -8.43484 |
| Darexaban | -8.4267 |
| Tenofovir disoproxil | -8.4124937 |
| Neratinib | -8.39573 |
| Ponatinib | -8.38035 |
| 6-(3-(Dimethylcarbamoyl)phenylsulfonyl)-4-(3-methoxyphenylamino)-8-methylquinoline-3-carboxamide | -8.32773 |
| 2'-Deoxy-N-(naphthalen-1-ylmethyl)guanosine 5'-(dihydrogen phosphate) | -8.31957 |
| 3-(5-{[4-(Aminomethyl)piperidin-1-Yl]methyl}-1h-Indol-2-Yl)quinolin-2(1h)-One | -8.30333 |
| (2R)-N-[2-[4-[5-[4-[(4-Acetamidophenyl)methoxy]-2,3-dichlorophenyl]-2-methylpyrazol-3-yl]piperidin-1-yl]-2-oxoethyl]-2-(diaminomethylideneamino)-4-methylpentanamide | -8.2941 |
| Butafenacil | -8.29337 |
| Nilotinib | -8.28767 |
| Gedatolisib | -8.27715 |
| N-(Sulfanylacetyl)tyrosylprolylmethioninamide | -8.27166 |
| Leucovorin | -8.2563314 |
| Asp3026 | -8.24956 |
| Methyl (1R,2S)-2-(hydroxycarbamoyl)-1-[[4-[(2-methylquinolin-4-yl)methoxy]phenyl]methyl]cyclopropane-1-carboxylate | -8.2435 |
| GW-493838 | -8.1977 |
| 2-({4-[4-(Pyridin-4-ylmethyl)-1H-pyrazol-3-yl]phenoxy}methyl)quinoline | -8.18944 |
| 2-({4-[(5-Chloro-1H-indol-2-YL)sulfonyl]piperazin-1-YL}carbonyl)thieno[3,2-B]pyridine 4-oxide | -8.17951 |
| Travoprost | -8.162818 |

| | |
|---|---|
| Hexamidine | -8.14578 |
| 4-(1,3-Benzodioxol-5-Yloxy)-2-[4-(1h-Imidazol-1-Yl)phenoxy]pyrimidine | -8.14297 |
| Sjg-136 | -8.12716 |
| (2S,3R,6S)-4-Oxo-6-[4-[(2-phenylquinolin-4-yl)methoxy]phenyl]-5-azaspiro[2.4]heptane-2-carboxylic acid | -8.10969 |
| ORG-25435 | -8.10411 |
| Squalene | -8.10209 |
| 6,7-Dimethoxy-4-[(3r)-3-(2-Naphthyloxy)pyrrolidin-1-Yl]quinazoline | -8.10006 |
| (5S)-5-[[4-[2-(5-Methyl-2-phenyl-1,3-oxazol-4-yl)ethoxy]-1-benzothiophen-7-yl]methyl]-1,3-thiazolidine-2,4-dione | -8.09299 |
| Asp-4058 | -8.08662 |
| (5S)-5-[[4-[3-(5-Methyl-2-phenyl-1,3-oxazol-4-yl)propanoyl]phenyl]methyl]-1,3-thiazolidine-2,4-dione | -8.02981 |
| Uridylyl-2'-5'-phospho-adenosine | -8.02227 |
| HKI-357 | -8.02173 |
| n-[4-[[Glutamic acid]-carbonyl]-benzene-sulfonyl-d-prolinyl]-3-amino-propanoic acid | -8.02126 |
| Grapiprant | -8.00985 |
| Dirlotapide | -8.00538 |
| AMG-131 | -7.98479 |
| Flumatinib | -7.9804 |
| Siponimod | -7.97261 |
| Bafetinib | -7.97087 |
| 1-((2-Hydroxyethoxy)methyl)-5-(3-(benzyloxy)benzyl)-6-hydroxypyrimidine-2,4(1H,3H)-dione | -7.96472 |
| (1-Hydroxy-1-phosphono-2-[1,1';4',1'']terphenyl-3-yl-ethyl)-phosphonic acid | -7.95892 |
| Toreforant | -7.95254 |
| Ceftriaxone | -7.94988 |
| Atecegatran metoxil | -7.94223 |
| N-(5-{[(2s)-4-Amino-2-(3-Chlorophenyl)butanoyl]amino}-1h-Indazol-3-Yl)benzamide | -7.93776 |
| Lumacaftor | -7.91532 |
| Alvelestat | -7.89843 |
| Aldose reductase-IN-1 | -7.89767 |
| Guanosine 5'-diphosphate 2':3'-cyclic monophosphate | -7.8961 |
| 5'-Guanosine-diphosphate-monothiophosphate | -7.89037 |
| 6-(12-Hydroxydodecyl)-2,3-dimethoxy-5-methyl-1,4-benzoquinone | -7.88111 |
| Dihydrofolic acid | -7.88086 |
| Danusertib | -7.87696 |
| Unii-642PS51324 | -7.87616 |
| Dasatinib | -7.87318 |
| [(3S)-3-(Dimethylamino)pyrrolidin-1-yl]-[4-[[5-fluoro-4-(2-methyl-3-propan-2-ylimidazol-4-yl)pyrimidin-2-yl]amino]phenyl]methanone | -7.83605 |
| Raltitrexed | -7.83305 |
| Glesatinib | -7.82904 |
| N,4-Dimethyl-3-[(1-phenyl-1H-pyrazolo[3,4-d]pyrimidin-4-yl)amino]benzamide | -7.82707 |
| 2,6-Dimethyl-1-(3-[3-methyl-5-isoxazolyl]-propanyl)-4-[2n-methyl-2h-tetrazol-5-yl]-phenol | -7.82541 |
| Tivozanib | -7.825 |
| Trifluorofurnesyl diphosphate | -7.82168 |

| | |
|---|---|
| 5-(7-(5-Hydro-4-methyl-2-oxazolyl)phenoxy)heptyl)-3-methyl isoxazole | -7.81605 |
| Norbixin | -7.81135 |
| Aminoquinuride | -7.80693 |
| Entinostat | -7.80107 |
| Pyrotinib | -7.79242 |
| 4-{[1-Methyl-5-(2-methyl-benzoimidazol-1-ylmethyl)-1H-benzoimidazol-2-ylmethyl]-amino}-benzamidine | -7.78962 |
| 3-[[3-Fluoro-2-(methylsulfamoylamino)pyridin-4-yl]methyl]-4-methyl-7-pyrimidin-2-yloxychromen-2-one | -7.77858 |
| Troriluzole | -7.77349 |
| Olaparib | -7.76574 |
| 1,3-Dimethyl-7-[2-[[(2R)-1-phenylpropan-2-yl]amino]ethyl]purine-2,6-dione | -7.76025 |
| Axitinib | -7.72446 |
| 5-{2-Fluoro-5-[3-(3-hydroxy-2-methoxycarbonyl-phenoxy)-propenyl]-phenyl}-isoxazole-3-carboxylic acid | -7.72172 |
| Atevirdine | -7.71884 |
| N-{3-[4-(3-Amino-propyl)-piperazin-1-YL]-propyl}-3-nitro-5-(galactopyranosyl)-beta-benzamide | -7.71813 |
| Flufenoxuron | -7.71614 |
| 4-Amino-8-(2-fluoro-6-methoxyphenyl)-N-propylcinnoline-3-carboxamide | -7.71045 |
| Teneligliptin | -7.70647 |
| Pexidartinib | -7.70575 |
| Cerdulatinib | -7.70386 |
| Phthalylsulfathiazole | -7.69539 |
| N-(4-Phenoxyphenyl)-2-[(Pyridin-4-Ylmethyl)amino]nicotinamide | -7.68645 |
| PTP1B Inhibitor | -7.68611 |
| Novobiocin | -7.68053 |
| HT-0712 | -7.67965 |
| (9E)-15-(2-Pyrrolidin-1-ylethoxy)-7,12,26-trioxa-19,21,24-triazatetracyclo[18.3.1.12,5.114,18]hexacosa-1(24),2,4,9,14,16,18(25),20,22-nonaene | -7.67722 |
| N-Cyclopropyl-4-methyl-3-[1-(2-methylphenyl)phthalazin-6-yl]benzamide | -7.67502 |
| Penamecillin | -7.66775 |
| Carboxyamidotriazole | -7.66609 |
| 3-[2,6,8-Trioxo-9-[(2S,3S,4R)-2,3,4,5-tetrahydroxypentyl]-3H-purin-7-yl]propyl dihydrogen phosphate | -7.66503 |
| (7R)-7-Phenyl-7-(2,4,5-trimethyl-3,6-dioxocyclohexa-1,4-dien-1-yl)heptanoic acid | -7.66483 |
| Losmapimod | -7.65774 |
| Alamifovir | -7.6565 |
| HG_7_85_01 | -7.65557 |
| 5-[3-(2-Methoxyphenyl)-1h-Pyrrolo[2,3-B]pyridin-5-yl]-n,n-dimethylpyridine-3-carboxamide | -7.65465 |
| Olmutinib | -7.65387 |
| 5-(4-Morpholin-4-YL-phenylsulfanyl)-2,4-quinazolinediamine | -7.6527 |
| CID 6914621 | -7.65228 |
| 1-(5-Chloro-6-(2-methylpropoxy)-3-pyridinyl)-3-methyl-N-(methylsulfonyl)-1H-indazole-5-carboxamide | -7.64941 |
| s-Benzyl-glutathione | -7.64403 |
| Simfibrate | -7.63094 |
| Etofamide | -7.62563 |

| | |
|---|---|
| Verdinexor | -7.62995 |
| 5-[[6-[2,4-Bis(trifluoromethyl)phenyl]pyridazin-3-yl]methyl]-2-(2-fluorophenyl)-1h-imidazo[4,5-c]pyridine | -7.62547 |
| S-23 | -7.62451 |
| N-[4-(2-Chlorophenyl)-1,3-dioxo-1,2,3,6-tetrahydropyrrolo[3,4-c]carbazol-9-yl]formamide | -7.61237 |
| Unii-XH93U6nije | -7.60956 |
| 2-[3-[(2S)-4,4-Difluoro-2-(pyrrolidine-1-carbonyl)pyrrolidin-1-yl]-3-oxopropyl]isoindole-1,3-dione | -7.60441 |
| N6-Isopentenyl-adenosine-5'-monophosphate | -7.60094 |
| Sonedenoson | -7.59855 |
| Lintitript | -7.59544 |
| 2-Hydroxy-5-({1-[(2-naphthyloxy)methyl]-3-oxoprop-1-enyl}amino)tyrosine | -7.59333 |
| ATP* | -7.59121 |
| Honokiol* | -6.99529 |
| hkh40a* | -6.66791 |
| VER-155008* | -6.81304 |
| Isoliquiritigenin* | -6.19445 |
| Epigallocatechin gallate* | -4.94584 |

*positive controls.

**Table 2S.** Results of the docking of DrugBank compounds onto GRP78 (SBD) (only molecules with lower docking scores than control region IV (C480-C488) from SARS-CoV-2 spike are presented).

| **Molecule name** | **Final docking score S (kcal/mol)** |
|---|---|
| Zilucoplan | -13.534038 |
| Obinepitide | -13.276237 |
| Corticorelin ovine triflutate | -13.069051 |
| CID 131704298 | -12.854046 |
| N-[(3E)-1-Oxo-3-hexenyl]Somatoliberin | -12.760726 |
| Tifuvirtide | -12.726995 |
| Cotadutide | -12.652015 |
| Semaglutide | -12.464309 |
| CID 118984461 | -12.390542 |
| Taspoglutide | -11.908224 |
| Adrenocorticotropic hormone 1-24 | -11.165439 |
| Adenosine-5'-ditungstate | -11.0066 |
| CID 16139342 | -10.820189 |
| Brimapitide | -10.814741 |
| CID 145994601 | -10.75272 |
| Albuvirtide | -10.431156 |

| | |
|---|---|
| Tetrastearoylcardiolipin | -10.306842 |
| Exendin (9-39) | -9.8784151 |
| Bivalirudin | -9.8586502 |
| Sinapultide | -9.7754955 |
| [2-Amino-3-[3-[2-[2-[3-[[2-[3-[4-[3-[[3-nitro-5-[3,4,5-trihydroxy-6-(hydroxymethyl)oxan-2-yl]oxybenzoyl]amino]propyl]piperazin-1-yl]propylamino]-3,4-dioxocyclobuten-1-yl]amino]propoxy]ethoxy]ethoxy]propylcarbamoyloxy]propyl] N-[3-[2-[2-[3-[[2-[3-[4-[3-[[3-nitro-5-[(2R,3R,4S,5R,6R)-3,4,5-trihydroxy-6-(hydroxymethyl)oxan-2-yl]oxybenzoyl]amino]propyl]piperazin-1-yl]propylamino]-3,4-dioxocyclobuten-1-yl]amino]propoxy]ethoxy]ethoxy]propyl]carbamate | -9.7283964 |
| Acyline | -9.5540953 |
| Edratide | -9.5526524 |
| Modimelanotide | -9.4725552 |
| (2R)-N-[(3R,6S,9S,15S,18R,21S,24R,27R,30S,33R,36R,39R,42R,45R,48S,49R)-24,42-Bis(3-aminopropyl)-27-benzyl-49-carbamoyl-3-(3-chloro-4-hydroxyphenyl)-21-[4-[(2S,3S,4S,5S,6R)-4,5-dihydroxy-6-(hydroxymethyl)-3-[(2R,3S,4S,5S,6R)-3,4,5-trihydroxy-6-(hydroxymethyl)oxan-2-yl]oxyoxan-2-yl]oxyphenyl]-18,39-bis[(1R)-1-hydroxyethyl]-30-[(1S)-1-hydroxyethyl]-15,33,36,45-tetrakis(4-hydroxyphenyl)-6-methyl-9-(2-methylpropyl)-2,5,8,11,14,17,20,23,26,29,32,35,38,41,44,47-hexadecaoxo-1-oxa-4,7,10,13,16,19,22,25,28,31,34,37,40,43,46-pentadecazacyclononatetracont-48-yl]-2-[[(2Z,4Z)-7-methylocta-2,4-dienoyl]amino]butanediamide | -9.3713093 |
| Sor-C13 | -9.3626308 |
| CID 145994612 | -9.294446 |
| alpha-[4-(1,1,3,3-Tetramethylbutyl)phenyl]-omega-hydroxy-poly(oxy-1,2-ethanediyl) | -9.2868958 |
| 2-[[9-[(2-Amino-4-methylsulfanylbutanoyl)amino]-39,62-bis(3-amino-3-oxopropyl)-24-benzyl-48,51,86,92-tetrakis(3-carbamimidamidopropyl)-17a,20a,33-tris(carboxymethyl)-80-[4-[6-[(2Z)-2-[(2E,4E,6E)-7-[1,1-dimethyl-3-(4-sulfobutyl)benzo[e]indol-3-ium-2-yl]hepta-2,4,6-trienylidene]-1,1-dimethylbenzo[e]indol-3-yl]hexanoylamino]butyl]-27,30-bis(1-hydroxyethyl)-74-[(4-hydroxyphenyl)methyl]-36-(1H-imidazol-4-ylmethyl)-45-methyl-8a-(2-methylpropyl)-12,42-bis(2-methylsulfanylethyl)-a,3,7a,10,10a,13,15a,18a,19,21a,22,25,28,31,34,37,40,43,46,49,52,61,64,70,73,76,79,82,85,88,91,94,97-tritriacontaoxo-2a,3a,6,7,12a,13a,56,57-octathia-2,6a,9a,11,14,16a,19a,20,22a,23,26,29,32,35,38,41,44,47,50,53,60,63,69,72,75,78,81,84,87,90,93,96,99-tritriacontazahexacyclo[57.41.10.84,54.421,77.014,18.065,69]docosahectane-5a-carbonyl]amino]-5-carbamimidamidopentanoic acid | -9.2015409 |
| Ularitide | -9.1868734 |
| [2-Amino-3-[2-[[2-[3-[4-[3-[[3-nitro-5-[3,4,5-trihydroxy-6-(hydroxymethyl)oxan-2-yl]oxybenzoyl]amino]propyl]piperazin-1-yl]propylamino]-3,4-dioxocyclobuten-1-yl]amino]ethylcarbamoyloxy]propyl] N-[2-[[2-[3-[4-[3-[[3-nitro-5-[(2R,3R,4S,5R,6R)-3,4,5-trihydroxy-6-(hydroxymethyl)oxan-2-yl]oxybenzoyl]amino]propyl]piperazin-1-yl]propylamino]-3,4-dioxocyclobuten-1-yl]amino]ethyl]carbamate | -9.1432199 |
| (2S)-N-[(2S)-1-[[(2S)-1-[[2-[[(2S)-4-Amino-1-[[(2S)-5-amino-1-[[(2S)-1-[[(2S)-1-[[(2S)-1-[[2-[[(2S)-1-[[(2S)-1-[[(2S)-1-amino-4-methylsulfanyl-1-oxobutan-2-yl]amino]-4-methyl-1-oxopentan-2-yl]amino]-3-(1H-imidazol-4-yl)-1- | -9.1206102 |

| | |
|---|---|
| oxopropan-2-yl]amino]-2-oxoethyl]amino]-3-methyl-1-oxobutan-2-yl]amino]-1-oxopropan-2-yl]amino]-3-(1H-indol-3-yl)-1-oxopropan-2-yl]amino]-1,5-dioxopentan-2-yl]amino]-1,4-dioxobutan-2-yl]amino]-2-oxoethyl]amino]-4-methyl-1-oxopentan-2-yl]amino]-5-carbamimidamido-1-oxopentan-2-yl]-2-[[(2S)-5-oxopyrrolidine-2-carbonyl]amino]pentanediamide | |
| (2R)-6-Amino-2-[[(2R)-2-[[(2R)-2-[[(2R)-2-[[(2R)-2-[[(2R,3R)-2-[[(2R)-2-[[(2R)-1-[(2R)-2-[[(2R)-2-[[(2R)-2-[[(2R,3R)-2-[[(2R)-2-[[(2R)-2-amino-3-(1H-indol-3-yl)propanoyl]amino]-3-(1H-indol-3-yl)propanoyl]amino]-3-hydroxybutanoyl]amino]-3-phenylpropanoyl]amino]-3-phenylpropanoyl]amino]-4-methylpentanoyl]pyrrolidine-2-carbonyl]amino]-3-hydroxypropanoyl]amino]-3-hydroxybutanoyl]amino]-4-methylpentanoyl]amino]-3-(1H-indol-3-yl)propanoyl]amino]-4-carboxybutanoyl]amino]-5-carbamimidamidopentanoyl]amino]hexanoic acid | -9.1110048 |
| Omiganan | -8.9104519 |
| Ganirelix | -8.7780027 |
| Triptorelin | -8.5557013 |
| Ozarelix | -8.5525694 |
| Melanotan-1 | -8.5197821 |
| Gramicidin D | -8.4410248 |
| 4F-Benzoyl-TN14003 | -8.3783236 |
| Compstatin analog peptide CP40 | -8.3450165 |
| Degarelix | -8.3361301 |
| [(3R)-1-[(2R,3R,4R,5S,6R)-2-[[(2R,3S,4R,5R,6R)-3-Hydroxy-4-[(3R)-3-hydroxydecoxy]-5-(3-oxotetradecanoylamino)-6-phosphonooxyoxan-2-yl]methoxy]-6-(methoxymethyl)-3-(3-oxotetradecanoylamino)-5-phosphonooxyoxan-4-yl]oxydecan-3-yl] (Z)-dodec-5-enoate | -8.2986946 |
| (2S)-2-[[4-[(2-Amino-4-oxo-3H-pteridin-6-yl)methylamino]benzoyl]amino]-3-[4-[2-[2-[3,3-dimethyl-5-sulfo-1-(4-sulfobutyl)indol-1-ium-2-yl]ethenyl]-6-[2-[3,3-dimethyl-5-sulfo-1-(4-sulfobutyl)indol-2-ylidene]ethylidene]cyclohexen-1-yl]oxyphenyl]propanoic acid | -8.2736235 |
| Fondaparinux | -8.1998796 |
| Immther | -8.1666164 |
| Abarelix | -8.0779819 |
| 3-Nitro-N-[3-[4-[3-[[2-[3-[4-[3-[[3-nitro-5-[(2R,3R,4S,5R,6R)-3,4,5-trihydroxy-6-(hydroxymethyl)oxan-2-yl]oxybenzoyl]amino]propyl]piperazin-1-yl]propylamino]-3,4-dioxocyclobuten-1-yl]amino]propyl]piperazin-1-yl]propyl]-5-[3,4,5-trihydroxy-6-(hydroxymethyl)oxan-2-yl]oxybenzamide | -7.9749308 |
| Daptomicina | -7.9554586 |
| Ceruletide | -7.8939037 |
| L-Phenylalaninamide, N2-(1-oxohexadecyl)-L-lysyl-L-lysyl-L-seryl-L-arginyl-L-alanyl-L-leucyl- | -7.8754039 |
| Somatostatin | -7.8446903 |
| Fertagyl | -7.7792234 |
| Tetradecanoyl-CoA | -7.7791991 |
| 2-(4-Amino-4,6-dimethyl-5-oxooxan-2-yl)oxy-22-(2-amino-2-oxoethyl)-5,15-dichloro-18,32,35,37-tetrahydroxy-19-[[4-methyl-2-(methylamino)pentanoyl]amino]-20,23,26,42,44-pentaoxo-48-[3,4,5-trihydroxy-6-(hydroxymethyl)oxan-2-yl]oxy-7,13-dioxa-21,24,27,41,43-pentazaoctacyclo[26.14.2.23,6.214,17.18,12.129,33.010,25.034,39]pentaconta-3,5,8,10,12(48),14,16,29(45),30,32,34(39),35,37,46,49-pentadecaene-40-carboxylic acid | -7.741888 |

| | |
|---|---|
| Sincalide | -7.729105 |
| Bradykinin | -7.6730123 |
| Rimiducid | -7.6237817 |
| Unii-YI2S0guq2C | -7.618494 |
| Coenzyme Q10 | -7.6177607 |
| Delparantag | -7.6103325 |
| (4S)-4-[[(2S)-2-[[(2S)-2-[[(2S)-2-[[(2S)-2-Acetamido-3-hydroxypropanoyl]amino]-3-(4-hydroxyphenyl)propanoyl]amino]-3-hydroxypropanoyl]amino]-4-methylsulfanylbutanoyl]amino]-5-[[1-[[(2S)-1-[[(2S)-1-[[(2S)-1-[[2-[[(2S)-6-amino-1-[(2S)-2-[[(2S)-1-amino-3-methyl-1-oxobutan-2-yl]carbamoyl]pyrrolidin-1-yl]-1-oxohexan-2-yl]amino]-2-oxoethyl]amino]-3-(1H-indol-3-yl)-1-oxopropan-2-yl]amino]-5-carbamimidamido-1-oxopentan-2-yl]amino]-1-oxo-3-phenylpropan-2-yl]amino]-3-(1H-imidazol-5-yl)-1-oxopropan-2-yl]amino]-5-oxopentanoic acid | -7.6015368 |
| Suramin | -7.5867271 |
| Antarelix | -7.5620089 |
| Bepecin | -7.5540962 |
| Sinapoyl coenzyme A | -7.5208478 |
| Triolein | -7.5046391 |
| Dotatate | -7.4802132 |
| Abt-510 | -7.4741888 |
| Bhsar-SP | -7.4709034 |
| Angiotensin II | -7.4678698 |
| Oncopore | -7.4600463 |
| Bambermycins | -7.4599528 |
| Ala-geninthiocin | -7.4431362 |
| alpha-GalCer | -7.4146681 |
| Palmitoyl tetrapeptide-7 | -7.3767581 |
| 3-[(1R,6R,9S,12S,15S,21S,24S,27S,30S,33R,36S,39S,45S,48S,53R)-53-[(2-Aminoacetyl)amino]-30-(2-amino-2-oxoethyl)-9-[(2S)-butan-2-yl]-36-(3-carbamimidamidopropyl)-6-carbamoyl-24,45-bis(carboxymethyl)-48-(hydroxymethyl)-27-[(4-hydroxyphenyl)methyl]-21-(1H-imidazol-5-ylmethyl)-8,11,14,20,23,26,29,32,35,38,44,47,50,52-tetradecaoxo-3,4,55,56-tetrathia-7,10,13,19,22,25,28,31,34,37,43,46,49,51-tetradecazatetracyclo[31.17.7.015,19.039,43]heptapentacontan-12-yl]propanoic acid | -7.3716264 |
| Deslorelin | -7.3678479 |
| Etelcalcetide | -7.3659992 |
| Uproleselan | -7.3155847 |
| Leuprorelin | -7.3120184 |
| (2R,3R,4S,5S,6R)-2-[(2R,3S,4R,5R,6S)-6-[(1R,4S,5S,6R)-4-[[(2R,3S,4S,5R,6R)-6-[(2R,3S,4R,5R,6R)-4,5-Dihydroxy-2-(hydroxymethyl)-6-[(2R,3S,4R,5R,6S)-4,5,6-trihydroxy-2-(hydroxymethyl)oxan-3-yl]oxyoxan-3-yl]oxy-4,5-dihydroxy-2-methyloxan-3-yl]amino]-5,6-dihydroxy-2-(hydroxymethyl)cyclohex-2-en-1-yl]oxy-4,5-dihydroxy-2-(hydroxymethyl)oxan-3-yl]oxy-6-(hydroxymethyl)oxane-3,4,5-triol | -7.2878718 |
| Bremelanotide | -7.2748389 |
| D-Glucopyranose, pentakis[3,4-dihydroxy-5-[(3,4,5-trihydroxybenzoyl)oxy]benzoate] | -7.2686925 |
| Mivacurium | -7.2632093 |

| | |
|---|---|
| Colistin sodium methanesulfonate | -7.2589989 |
| Cibinetide | -7.2571716 |
| Goserelin | -7.2503462 |
| Vapreotide | -7.2494888 |
| Glutathionylspermidine disulfide | -7.2469893 |
| Tat-BP | -7.2457705 |
| [2-Amino-3-[3-[4-[3-[[3-nitro-5-[3,4,5-trihydroxy-6-(hydroxymethyl)oxan-2-yl]oxybenzoyl]amino]propyl]piperazin-1-yl]propylcarbamoyloxy]propyl] N-[3-[4-[3-[[3-nitro-5-[(2R,3R,4S,5R,6R)-3,4,5-trihydroxy-6-(hydroxymethyl)oxan-2-yl]oxybenzoyl]amino]propyl]piperazin-1-yl]propyl]carbamate | -7.2409163 |
| Setmelanotide | -7.2327309 |
| CID 17753951 | -7.2154012 |
| Depreotide | -7.2144785 |
| Nafarelin | -7.2102528 |
| 1-Palmitoyl-2-oleoyl-sn-glycero-3-phosphoglycerol | -7.2046738 |
| Eritoran | -7.1976666 |
| Lanreotide acetate | -7.1871324 |
| Bacitracin A | -7.1833935 |
| CoA-S-acetyl 5-bromotryptamine | -7.1813159 |
| Semapimod | -7.1751194 |
| Terlipressin | -7.1704521 |
| Notomycin A1 | -7.1515746 |
| (2R)-N-[(2S)-6-Amino-1-anilino-1-oxohexan-2-yl]-2-[[2-[(2S)-2-[[(2S)-6-amino-1-anilino-1-oxohexan-2-yl]carbamoyl]hexyl]hydrazinyl]methyl]hexanamide | -7.1362066 |
| (R)-3-Hydroxydecanoyl-CoA | -7.1291924 |
| Arylomycin A2 | -7.1237774 |
| Inhibitor bea409 | -7.123332 |
| Reidispongiolide A | -7.115509 |
| Lauroyl-CoA | -7.1137977 |
| Trierucin | -7.1008019 |
| Labradimil | -7.1003852 |
| C24:1 Sulfatide | -7.0981321 |
| Ubiquinol-10 | -7.0977497 |
| Unii-rwy5U5kzv3 | -7.0745702 |
| Brilacidin | -7.074276 |
| Etarfolatide | -7.062871 |
| Doxacurium | -7.0600328 |
| Colistina | -7.0597138 |
| (3S,6S,12S,15S,18S,21S,24R,27S)-3,6-Dibenzyl-12-[(2R)-butan-2-yl]-4,16,22-trimethyl-24-[(2R)-3-methylbutan-2-yl]-18-(2-methylpropyl)-9,15,21-tri(propan-2-yl)-13-oxa-1,4,7,10,16,19,22,25-octazabicyclo[25.3.0]triacontane-2,5,8,11,14,17,20,23,26-nonone | -7.0276241 |
| Venetoclax | -7.0154495 |
| Candicidin D | -6.9966493 |
| Telavancin | -6.9900298 |
| Triglu-5-formyl-tetrahydrofolate | -6.9813905 |
| Ethylhexyl triazone | -6.9788446 |
| L-Carnitinyl-CoA | -6.9501667 |
| Dipalmitoylphosphatidylglycerol | -6.9480577 |
| Sulfogalactoceramide | -6.9408188 |

| | |
|---|---|
| Dimyristoylphosphatidylglycerol | -6.9336691 |
| Octreotide acetate | -6.9301686 |
| Tenapanor | -6.9041467 |
| 2-[[(4S)-4-[[(4S)-4-[[(4S)-4-[[4-[2-(2-Amino-4-oxo-3,7-dihydropyrrolo[2,3-d]pyrimidin-5-yl)ethyl]benzoyl]amino]-4-carboxybutanoyl]amino]-4-carboxybutanoyl]amino]-4-carboxybutanoyl]amino]pentanedioic acid | -6.9035096 |
| 2-[[(2R)-2,3-Bis[[(9Z,12Z)-octadeca-9,12-dienoyl]oxy]propoxy]-hydroxyphosphoryl]oxyethyl-trimethylazanium | -6.9006743 |
| Angiotensin (1-7) | -6.8859501 |
| Nona-arginine | -6.881742 |
| DSPC;L-beta,gamma-Distearoyl-alpha-lecithin | -6.8807964 |
| Demoxytocin | -6.8710155 |
| 5-Mercaptoethanol-2-decenoyl-coenzyme A | -6.8692455 |
| 1,2-Dioleoyl-sn-glycero-3-phosphocholine(1+) | -6.8565731 |
| Velpatasvir | -6.852726 |
| Voclosporin | -6.844852 |
| Proglumetacin | -6.8427505 |
| Unii-J1J4P3pqzd | -6.832921 |
| Sugammadex | -6.8125958 |
| Selepressin | -6.8108587 |
| (3S)-4-[[(3S,6R,12S,15S,18S,21R,24S,28S,30R)-18-(4-Aminobutyl)-6-(1-carboxyethyl)-15-[carboxy(hydroxy)methyl]-12-(carboxymethyl)-25,30-dimethyl-2,5,8,11,14,17,20,23,27-nonaoxo-3,21-di(propan-2-yl)-1,4,7,10,13,16,19,22,26-nonazabicyclo[26.3.0]hentriacontan-24-yl]amino]-2-methyl-3-[[(2E,4Z)-8-methyldeca-2,4-dienoyl]amino]-4-oxobutanoic acid | -6.8021159 |
| 2-[[4-[[(2S)-3-Methyl-1-oxo-1-[(2S)-2-[[(3S)-1,1,1-trifluoro-4-methyl-2-oxopentan-3-yl]carbamoyl]pyrrolidin-1-yl]butan-2-yl]carbamoyl]benzoyl]amino]acetic acid | -6.8007655 |
| Aclerastide | -6.7991471 |
| 5-Hydroxyferuloyl-CoA | -6.79245 |
| Ipamorelin | -6.7911024 |
| Friulimycin B | -6.7760978 |
| CID 5289562 | -6.7739582 |
| Anatibant | -6.76813 |
| Angiotensinamide | -6.7556543 |
| Carfilzomib | -6.7467966 |
| Murepavadin | -6.7444425 |
| 2-[4-[(2S)-2-Acetamido-3-oxo-3-[[(3S)-2-oxo-1-[(4-phenylphenyl)methyl]azepan-3-yl]amino]propyl]-2-methoxycarbonylphenyl]-2-fluoropropanedioic acid | -6.7250972 |
| N-(2-Hydroxyoctadecanoyl)-4R-hydroxysphinganine | -6.7219591 |
| Cetrorelix | -6.7210383 |
| Reactive Red 6 hapten | -6.719985 |
| Alisporivir | -6.7160454 |
| Ddavp | -6.7113504 |
| 3-Thiaoctanoyl-coenzyme A | -6.710319 |
| Bis(gamma-glutamyl-cysteinyl-glycinyl)spermidine | -6.7033076 |
| Soblidotin | -6.6945066 |
| Nicotinamide-Adenine-Dinucleotide | -6.6828265 |
| Dadle | -6.6812816 |

| | |
|---|---|
| [4-[(2S)-2-Acetamido-3-[[(5S)-3-carbamoyl-2-(cyclohexylmethoxy)-6,7,8,9-tetrahydro-5H-benzo[7]annulen-5-yl]amino]-3-oxopropyl]-2-phosphonophenyl]phosphonic acid | -6.6811619 |
| Pibrentasvir | -6.67345 |
| Lodenafil carbonate | -6.6667376 |
| Valspodar | -6.6489363 |
| (2S)-N-[(2R)-1-[[(2S)-1-Amino-6-(diaminomethylideneamino)-1-oxohexan-2-yl]amino]-3-(4-hydroxyphenyl)-1-oxopropan-2-yl]-1-[(2R)-3-cyclohexyl-2-[[4-[(diaminomethylideneamino)methyl]cyclohexanecarbonyl]amino]propanoyl]azetidine-2-carboxamide | -6.648747 |
| Pasireotide | -6.6476345 |
| Flavin adenine dinucleotide | -6.6392889 |
| (3S)-4-[[(3S,6R,12S,15S,18S,21R,24S,28S,30R)-18-(4-Aminobutyl)-6-(1-carboxyethyl)-15-[carboxy(hydroxy)methyl]-12-(carboxymethyl)-25,30-dimethyl-2,5,8,11,14,17,20,23,27-nonaoxo-3,21-di(propan-2-yl)-1,4,7,10,13,16,19,22,26-nonazabicyclo[26.3.0]hentriacontan-24-yl]amino]-2-methyl-3-[[(2E,4Z)-8-methylnona-2,4-dienoyl]amino]-4-oxobutanoic acid | -6.6375017 |
| Temsirolimus | -6.6330647 |
| Crocin | -6.6299481 |
| Iscotrizinol | -6.6291451 |
| Cobicistat | -6.6274652 |
| Monogalactosyl-diacylglycerol | -6.6216884 |
| 4-[[(2S)-2-[[(2R)-2-[[(2R)-3-(1-Benzothiophen-3-yl)-2-(piperidine-4-carbonylamino)propanoyl]amino]-3-(1H-indol-3-yl)propanoyl]amino]-3-phenylpropanoyl]amino]piperidine-4-carboxamide | -6.6209373 |
| DI-Stearoyl-3-SN-phosphatidylcholine | -6.6160131 |
| Folate-FITC | -6.608109 |
| Boc-gamma-D-glu-L-lys(cbz)-D-boroala | -6.6065769 |
| DMPC;L-beta,gamma-Dimyristoyl-alpha-lecithin | -6.60566 |
| Ioglycamic Acid | -6.6030331 |
| (2S)-2-[[(2S)-2-[[(2S)-2-[[(2S,3R)-2-[[(2S)-2-[[(2S)-2-[[(2S)-2-[[(2S)-2-[[(2S)-2-[(2-Acetamidoacetyl)amino]propanoyl]amino]-5-amino-5-oxopentanoyl]amino]-3-phenylpropanoyl]amino]-3-hydroxypropanoyl]amino]-6-aminohexanoyl]amino]-3-hydroxybutanoyl]amino]propanoyl]amino]propanoyl]amino]-6-aminohexanoic acid | -6.5977979 |
| Ombitasvir | -6.5924101 |
| adenylate-3'-phosphate-[[2'-deoxy-uridine-5'-phosphate]-3'-phosphate] | -6.57637 |
| 4-[4-Carboxybutyl-[2-[(18S,25S,35S)-35-[(S)-hydroxy(phenyl)methyl]-28-(methoxymethyl)-21-methyl-18-[2-(methylamino)-2-oxoethyl]-16,23,30,33-tetraoxo-25-propan-2-yl-3,13,20,27,37-pentathia-7,17,24,31,34,39,40,41,42,43-decazaheptacyclo[34.2.1.12,5.112,15.119,22.126,29.06,11]tritetraconta-1(38),2(43),4,6(11),7,9,12(42),14,19(41),21,26(40),28,36(39)-tridecaen-8-yl]-1,3-thiazol-4-yl]carbamoyl]oxycyclohexane-1-carboxylic acid | -6.5604935 |
| Hexapeptide 3 | -6.5602956 |
| Anidulafungin | -6.5572481 |
| Methacrylyl-CoA | -6.5532589 |
| 4-amino-n-{4-[2-(2,6-dimethyl-phenoxy)-acetylamino]-3-hydroxy-1-isobutyl-5-phenyl-pentyl}-benzamide | -6.5443 |

| | |
|---|---|
| 8-[4-(2-Butoxyethoxy)phenyl]-1-(2-methylpropyl)-N-[4-[(3-propylimidazol-4-yl)methylsulfinyl]phenyl]-3,4-dihydro-2H-1-benzazocine-5-carboxamide | -6.5426102 |
| Micafungin | -6.534081 |
| Deslanoside | -6.5284748 |
| Saralasin | -6.5260315 |
| Rilapladib | -6.5220246 |
| 2-[2-[[2-[[(2R)-1-[[(4R,7S,10S,13R,16S,19R)-10-(4-Aminobutyl)-16-benzyl-4-[[(2R,3R)-1,3-dihydroxybutan-2-yl]carbamoyl]-7-[(1R)-1-hydroxyethyl]-13-(1H-indol-3-ylmethyl)-6,9,12,15,18-pentaoxo-1,2-dithia-5,8,11,14,17-pentazacycloicos-19-yl]amino]-1-oxo-3-phenylpropan-2-yl]amino]-2-oxoethyl]-(carboxymethyl)amino]ethyl-[2-[bis(carboxymethyl)amino]ethyl]amino]acetic acid | -6.5151515 |
| [(2S)-2-[(Z)-Hexadec-9-enoyl]oxy-3-[hydroxy(2-hydroxyethoxy)phosphoryl]oxypropyl] (Z)-octadec-9-enoate | -6.5102944 |
| DI-Stearoyl-3-SN-phosphatidylethanolamine | -6.5082421 |
| gliquidone | -6.50701 |
| Unii-19ztz9YC4O | -6.5061979 |
| Unii-5I2gjw5Z2Q | -6.5046973 |
| Zeaxanthin | -6.5043249 |
| 4SC-203 | -6.50353 |
| Priftin | -6.4999089 |
| Neladenoson bialanate | -6.4957514 |
| 2-Octaprenylphenol | -6.4947829 |
| Elamipretide | -6.492218 |
| CoA-s-acetyl tryptamine | -6.4837537 |
| (E)-N-[4-[3-Chloro-4-[(3-fluorophenyl)methoxy]anilino]-3-cyano-7-ethoxyquinolin-6-yl]-4-(dimethylamino)but-2-enamide | -6.4814477 |
| 3-Hydroxybutyryl-coenzyme A | -6.4812236 |
| N-Acetyl-N-[1-(1,1'-biphenyl-4-ylmethyl)-2-oxoazepan-3-YL]-3-formyl-O-phosphonotyrosinamide | -6.4790406 |
| Unii-qmh3V8vspb | -6.4690313 |
| 2-[[2-Hexadecanoyloxy-3-[(Z)-octadec-9-enoyl]oxypropoxy]-hydroxyphosphoryl]oxyethyl-trimethylazanium | -6.4652758 |
| Merotocin | -6.4613204 |
| Clorobiocin | -6.4612813 |
| Itacitinib | -6.46057 |
| Vancomycin | -6.4546638 |
| Uridylyl-2'-5'-phospho-adenosine | -6.4519858 |
| [[(2R,3S,4R,5R)-3,4-Dihydroxy-5-(6-iminopurin-9-ium-9-yl)oxolan-2-yl]methoxy-hydroxyphosphoryl] [(2R,3S,4S)-5-[7,8-dimethyl-5-(2-methylpropanoyl)-2,4-dioxo-1H-benzo[g]pteridin-10-yl]-2,3,4-trihydroxypentyl] hydrogen phosphate | -6.4514418 |
| 2-Ethoxyethyl (2s,3s)-4-((s)-2-benzyl-3-oxo-4-((3ar,8r,8as)-2-oxo-3,3a,8,8a-tetrahydro-2h-indeno[1,2-d]oxazol-8-yl)-2,3-dihydro-1h-pyrrol-2-yl)-3-hydroxy-1-phenylbutan-2-ylcarbamate | -6.45052 |
| Larazotide | -6.4498482 |
| Valinomicin | -6.4474573 |
| 2-(L-Phenylalanine)-8-L-lysinevasopressin | -6.4396729 |
| Odalasvir | -6.4347672 |
| Timcodar | -6.4333954 |

| | |
|---|---|
| Fasitibant | -6.4295049 |
| Trypan Blue free acid | -6.4288964 |
| 4-(N,N-Dimethylamino)cinnamoyl-CoA | -6.4194269 |
| 3,8-Diamino-6-phenyl-5-[6-[1-[2-[(1,2,3,4-tetrahydro-9-acridinyl)amino]ethyl]-1h-1,2,3-triazol-4-yl]hexyl]-phenanthridinium | -6.41461 |
| Denufosol | -6.4072843 |
| 4-{[(6S,9S,9aS)-1-(Benzylcarbamoyl)-2,9-dimethyl-4,7-dioxo-8-(8-quinolinylmethyl)octahydro-2H-pyrazino[2,1-c][1,2,4]triazin-6-yl]methyl}phenyl dihydrogen phosphate | -6.40557 |
| Efonidipine | -6.405437 |
| Fozivudine tidoxil | -6.4046016 |
| Difelikefalin | -6.398294 |
| N,N-[2,5-O-Dibenzyl-glucaryl]-DI-[valinyl-aminomethanyl-pyridine] | -6.3966646 |
| CID 131704254 | -6.3933682 |
| [(3E,5E,8R,9S,10R,11R,14S,15E,18R,20R,21E,24S)-24-[(E,2S,3S,4S,7R,8S,9R,10R)-9-Acetyloxy-7-[(2S)-2-(dimethylamino)propanoyl]oxy-12-[formyl(methyl)amino]-3-hydroxy-4,8,10-trimethyldodec-11-en-2-yl]-10-hydroxy-14,20-dimethoxy-9,11,15,18-tetramethyl-2-oxo-1-oxacyclotetracosa-3,5,15,21-tetraen-8-yl] (2S)-2-(dimethylamino)-3-methoxypropanoate | -6.3907189 |
| Ibodutant | -6.390193 |
| Telmisartan | -6.3899698 |
| Talactoferrin alpha | -6.3838325 |
| CID 131704228 | -6.3835149 |
| Menaquinone-7 | -6.3804426 |
| Brostallicin | -6.3760753 |
| Foxy-5 | -6.3707175 |
| N-(Ethylsulfonyl)-5-propoxy-L-tryptophyl-N~1~-{4-[amino(imino)methyl]benzyl}-L-glutamamide | -6.3693395 |
| 1-[(2R,3R,4S,5R)-5-[[[[(2R,3S,4R,5R)-5-(6-aminopurin-9-yl)-3,4-dihydroxy-tetrahydrofuran-2-yl]methoxy-hydroxy-phosphoryl]oxy-hydroxy-phosphoryl]oxymethyl]-3,4-dihydroxy-tetrahydrofuran-2-yl]pyridin-1-ium-3-carboxylic acid | -6.36784 |
| Olcegepant | -6.3647766 |
| 6-Phenyl-5-[6-[1-[2-(1,2,3,4-tetrahydroacridin-9-ylamino)ethyl]triazol-4-yl]hexyl]phenanthridin-5-ium-3,8-diamine | -6.3647103 |
| [2,4,6-Triisopropyl-phenylsulfonyl-l-[3-amidino-phenylalaninyl]]-n'-β-alaninyl-piperazine | -6.35334 |
| Reversin 121 | -6.3508687 |
| Manidipine | -6.3480783 |
| Selatogrel | -6.3477368 |
| Astaxanthin | -6.34728 |
| Cellulose hydroxyethylate | -6.340929 |
| 1,2-Dipalmitoyl-sn-glycero-3-phosphoethanolamine | -6.3409286 |
| Adamantane-1-carboxylic acid-5-dimethylamino-naphthalene-1-sulfonylamino-octyl-amide | -6.3398166 |
| 2-[4-[(2S)-3-[4-(3-Hydroxy-2-methoxycarbonylphenoxy)butylamino]-3-oxo-2-(prop-2-enoxycarbonylamino)propyl]-N-oxaloanilino]benzoic acid | -6.3295193 |
| Bietaserpine | -6.3292842 |
| Granotapide | -6.3289695 |

| | |
|---|---|
| (2R)-N-[2-[4-[5-[4-[(4-Acetamidophenyl)methoxy]-2,3-dichlorophenyl]-2-methylpyrazol-3-yl]piperidin-1-yl]-2-oxoethyl]-2-(diaminomethylideneamino)-4-methylpentanamide | -6.3274131 |
| 1-Palmitoyl-2-linoleoyl-3-acetyl-rac-glycerol | -6.3268423 |
| Ruzasvir | -6.3249669 |
| Bosentan | -6.3217754 |
| Muraglitazar | -6.3117418 |
| NADP nicotinamide-adenine-dinucleotide phosphate | -6.3117328 |
| Elobixibat | -6.3088441 |
| (S)-4-Phenyl-1-[3-(3-pyridyl)propyl]butyl N-[difluoro(3,4,5-trimethoxy)acetyl]-L-pipecolate | -6.3084021 |
| Cilofexor | -6.3075676 |
| Motuporin | -6.3067598 |
| CID 53340771 | -6.2961597 |
| Sitravatinib | -6.2925801 |
| Hydrocortisone cypionate | -6.28916 |
| Lifitegrast | -6.28518 |
| N-n-Butyl-N-methyl-11-(3,17beta-dihydroxyestra-1,3,5(10)-trien-7alpha-yl)undecanamide | -6.2850342 |
| Ethyl (E,4S)-4-[[(2S)-2-[[(2S)-2-[[(2S)-2-(furan-3-carbonylamino)-3-methylbutanoyl]amino]-3-methylbutanoyl]amino]-4-methylpentanoyl]amino]-5-[(3S)-2-oxopyrrolidin-3-yl]pent-2-enoate | -6.284081 |
| Fenebrutinib | -6.2834244 |
| Region IV (C480-C488) of SARS-CoV-2 spike* | -6.27480 |

*positive control.
